# Variable Smoothing for Weakly Convex Composite Functions

**DOI:** 10.1007/s10957-020-01800-z

**Published:** 2021-02-08

**Authors:** Axel Böhm, Stephen J. Wright

**Affiliations:** 1 Faculty of Mathematics, University of Vienna, Oskar-Morgenstern-Platz 1, 1090 Vienna, Austria; 2 Computer Sciences Department and Wisconsin Institute for Discovery, University of Wisconsin-Madison, 1210 W. Dayton St, Madison, WI 53706 USA

**Keywords:** Variable smoothing, Weakly convex, Composite minimization

## Abstract

We study minimization of a structured objective function, being the sum of a smooth function and a composition of a weakly convex function with a linear operator. Applications include image reconstruction problems with regularizers that introduce less bias than the standard convex regularizers. We develop a variable smoothing algorithm, based on the Moreau envelope with a decreasing sequence of smoothing parameters, and prove a complexity of ${\mathcal {O}}(\epsilon ^{-3})$ to achieve an $\epsilon$-approximate solution. This bound interpolates between the ${\mathcal {O}}(\epsilon ^{-2})$ bound for the smooth case and the ${\mathcal {O}}(\epsilon ^{-4})$ bound for the subgradient method. Our complexity bound is in line with other works that deal with structured nonsmoothness of weakly convex functions.

## Introduction

We study minimization of the sum of a smooth function and a nonsmooth, weakly convex function composed with a linear operator. The case in which the nonsmooth regularizer is convex has been studied extensively; see [1, 2]. Weakly convex functions (which can be expressed as the difference between a convex function and a quadratic) share some properties with convex functions but include many interesting nonconvex cases, as we discuss in Sect. 2.1. For example, any smooth function with a uniformly Lipschitz continuous gradient is a weakly convex function.

Our approach makes use of a smooth approximation of the weakly convex function known as the *Moreau envelope*, parametrized by a positive scalar $\mu$. Since evaluation of the gradient of the Moreau envelope is obtained by applying a proximal operator to the function, our method is suitable for problems where this proximal operator can be evaluated at reasonable cost. Our method requires ${\mathcal {O}}(\epsilon ^{-3})$ iterations to obtain an $\epsilon$-approximate stationary point.

The remainder of the paper is organized as follows. Section 2 is concerned with other problem formulations related to ours and describes specific problems with weakly convex regularizers. In Sect. 3, we give the necessary mathematical preliminaries including a detailed discussion about the notion of stationarity we use. Section 4 describes our approach and its convergence properties. In Sect. 5, we highlight the difference between the variable smoothing technique and a simple proximal gradient approach, for the case in which the linear operator is not present in the weakly smooth term.

## Problem Class and Algorithmic Approach

The problem we address in this paper has the form

1 $$\begin{aligned} \min _{x \in {\mathbb {R}}^d} \, F(x) := h(x) + g(Ax), \end{aligned}$$

for a smooth function $h:{\mathbb {R}}^d \rightarrow {\mathbb {R}}$, a weakly convex function $g:{\mathbb {R}}^n \rightarrow {\mathbb {R}}$ (generally nonsmooth) and a matrix $A\in {\mathbb {R}}^{n \times d}$. For some $\rho \ge 0$, we say that

$$\begin{aligned} g:{\mathbb {R}}^n \rightarrow {\overline{{\mathbb {R}}}} \text{ is } \rho -{\text {weakly convex if}} \;\; g + \frac{\rho }{2} \Vert \cdot \Vert ^2 \; \text{ is } \text{ convex. } \end{aligned}$$

When *g* is a smooth function with a uniformly Lipschitz continuous gradient, with Lipschitz constant *L*, then *g* is weakly convex with $\rho =L$. Other interesting weakly convex functions are discussed in Sect. 2.1.

The *Moreau envelope* $g_{\mu }$ is a smooth approximation of *g*, parametrized by a positive scalar $\mu$. The Moreau envelope and the closely related proximal operator are defined as follows.

### Definition 2.1

For a proper, $\rho$-weakly convex and lower semicontinuous function $g: {\mathbb {R}}^n \rightarrow {\overline{{\mathbb {R}}}}$, the Moreau envelope of *g* with the parameter $\mu \in ]0,\rho ^{-1}[$ is the function from ${\mathbb {R}}^n$ to ${\mathbb {R}}$ defined by

$$\begin{aligned} g_{\mu }(y) := \inf _{z \in {\mathbb {R}}^n} \left\{ g(z) + \frac{1}{2\mu }\Vert z-y \Vert ^2\right\} . \end{aligned}$$

The proximal operator of the function $\mu g$ is the ${{\,\mathrm{arg\,min}\,}}$ of the right-hand side in this definition, that is,

$$\begin{aligned} {\text {prox}}^{}_{\mu g}\left( y \right) := {\mathop {{{\,\mathrm{arg\,min}\,}}}\limits _{z \in {\mathbb {R}}^n}}\left\{ g(z) + \frac{1}{2\mu }\Vert z-y \Vert ^2\right\} {= {\mathop {{{\,\mathrm{arg\,min}\,}}}\limits _{z \in {\mathbb {R}}^n}}\left\{ \mu g(z) + \frac{1}{2}\Vert z-y \Vert ^2\right\} .} \end{aligned}$$

Note that ${\text {prox}}^{}_{\mu g}\left( y \right)$ is defined *uniquely* by this formula, because the function being minimized is strongly convex. We describe in Lemma 3.1 the relationship between $\nabla g_{\mu }(y)$ and ${\text {prox}}^{}_{\mu g}\left( y \right)$, which is key to our algorithm.

Steps of our algorithm have the form

$$\begin{aligned} x \leftarrow x - \gamma \nabla (h + g_{\mu } \circ A)(x), \end{aligned}$$

for some step length $\gamma$. Accelerated versions of these approaches have been proposed for convex problems in [3–5]. The use of acceleration makes the analysis more complicated than for the gradient case; see [6, 7].

### Composite Problems

We discuss several instances of problems of the form (1).

*Regularization with* $\Vert \cdot \Vert _1$ *(LASSO).* Functions that are “sharp” around zero have a long history as regularizers that induce sparsity in the solution vector *x*. Foremost among such functions is the vector norm $\Vert \cdot \Vert _1$, which is used for example in sparse least-squares regression (also known as LASSO [8]):

2 $$\begin{aligned} \min _x \, \frac{1}{2} \Vert Bx - b \Vert ^2 + \Vert x \Vert _1. \end{aligned}$$

This formulation is convex and forms a special case of (1) in which *A* is given by the identity. Regularization with the norm $\Vert \cdot \Vert _1$ is used also in logistic regression [9].

*Other Convex Regularizers.* The case of problems (1) in which *g* is nonsmooth and *convex* (with possible smooth and/or nonsmooth additive terms) has received a great deal of attention in the literature on convex optimization and applications; see for example [1, 4, 10–12]. The most notable applications are found in inverse problems involving images. In particular, discrete (an)isotropic *Total Variation (TV)* denoising has the form

$$\begin{aligned} \min _x \, \frac{1}{2} \Vert x-b \Vert ^2 + \Vert \nabla x \Vert _1, \end{aligned}$$

where *b* is the observed (noisy) image and $\nabla$ denotes the discretized gradient in two or three dimensions. TV deblurring problems have the form

$$\begin{aligned} \min _x \, \frac{1}{2} \Vert Bx-b \Vert ^2 + \Vert \nabla x \Vert _1, \end{aligned}$$

where *B* is the blurring operator; see [1, 13].

Other examples of convex problems of the form (1) include generalized convex feasibility [4] and support vector machine classification [5]. A typical formulation of the latter problem has $h(x) = (\lambda /2) \Vert x\Vert ^2$ and $g(Ax) = \sum _{i=1}^n \phi (y_i a_i^T x)$, where $\phi (s) = \max \{-s,0\}$ is the hinge loss and the rows of *A* are $y_i a_i^T$, $i=1,2, \ldots n$, where $(y_i,a_i) \in \{-1,1\} \times {\mathbb {R}}^d$ are the training points and their labels.

*Weakly Convex Regularizers.* The use of the $\ell _1$ regularizer in (2) tends to depress the magnitude of nonzero elements of the solution, resulting in *bias*. This phenomenon is a consequence of the fact that the proximal operator of the 1-norm, often called the *soft thresholding operator*, does not approach the identity even for large arguments. For this reason, nonconvex alternatives to $\Vert \cdot \Vert _1$ are often used to reduce bias. These include $\ell _p$-norms (with $0<p<1$) which are not weakly convex, and several weakly convex regularizers, which we now describe. The *minimax concave penalty (MCP)*, introduced in [14] and used in [15, 16], is a family of functions $r_{\lambda ,\theta }:{\mathbb {R}}\rightarrow {\mathbb {R}}_+$ involving two positive parameters $\lambda$ and $\theta$, and defined by

$$\begin{aligned} r_{\lambda ,\theta }(x) := {\left\{ \begin{array}{ll} \lambda \vert x \vert - \frac{x^2}{2 \theta }, &{} \vert x \vert \le \theta \lambda ,\\ \frac{\theta \lambda ^2}{2}, &{} \text {otherwise}. \end{array}\right. } \end{aligned}$$

(Note that this function satisfies the definition of $\rho$-weak convexity with $\rho = \theta ^{-1}$.) The proximal operator of this function (called *firm threshold* in [17]) can be written in the following closed form when $\theta >\gamma$:

$$\begin{aligned} {\text {prox}}^{}_{\gamma r_{\lambda ,\theta }}\left( x \right) = {\left\{ \begin{array}{ll} 0, &{} \vert x \vert < \gamma \lambda , \\ \frac{x- \lambda \gamma {{\,\mathrm{sgn}\,}}(x)}{ 1-({\gamma }/{\theta })}, &{} \gamma \lambda \le \vert x \vert \le \theta \lambda , \\ x , &{} \vert x \vert > \theta \lambda . \end{array}\right. } \end{aligned}$$

The *fractional penalty function* (cf. [16, 18]) $\phi _a:{\mathbb {R}}\rightarrow {\mathbb {R}}_+$ (for parameter $a>0$) is

$$\begin{aligned} \phi _a(x) := \frac{\vert x \vert }{1 + a \vert x \vert /2 }. \end{aligned}$$

The *smoothly clipped absolute deviation (SCAD)* [19] (cf. [16]) is defined for parameters $\lambda >0$ and $\theta >2$ as follows:

$$\begin{aligned} r_{\lambda ,\theta }(x) = {\left\{ \begin{array}{ll} \lambda \vert x \vert , &{} \vert x \vert \le \lambda , \\ \frac{-x^2+2\theta \lambda \vert x \vert - \lambda ^2}{2(\theta -1)}, &{} \lambda < \vert x \vert \le \theta \lambda , \\ \frac{(\theta +1)\lambda ^2}{2}, &{} \vert x \vert > \theta \lambda . \end{array}\right. } \end{aligned}$$

(This function is ${(\theta -1)}^{-1}$-weakly convex.)

Since these functions approach (or attain) a finite value as their argument grows in magnitude, they do not introduce as much bias in the solution as does the $\ell _1$ norm, and their proximal operators approach the identity for large arguments.

The regularizers of this section, and the convex $\Vert \cdot \Vert$ regularizer, have been used mostly in the simple additive setting

$$\begin{aligned} \min _{x \in {\mathbb {R}}^d} \, h(x) + g(x) \end{aligned}$$

for a smooth data fidelity term *h* and nonsmooth regularizer *g*, for example in least squares or logistic regression [15] and compressed sensing (cf. [17]).

*Weakly convex composite losses* The use of weakly convex functions composed with linear operators has been explored in the robust statistics literature. An early instance is the *Tukey biweight* function [20], in which *g*(*Ax*) has the form

3 $$\begin{aligned} g(Ax) = \sum _{i=1}^n \phi (A_{i\cdot }x-b_i), \quad \text{ where } \; \phi (\theta ) = \frac{\theta ^2}{1+\theta ^2}, \end{aligned}$$

where $A_{i\cdot }$ denotes the *i*th row of *A*. This function behaves like the usual least-squares loss when $\theta ^2 \ll 1$ but asymptotes at 1. It is $\rho$-weakly convex with $\rho =6$.

A slightly different definition of the Tukey biweight function appears in [21, Section 2.1]. This same reference also mentions another nonconvex loss function, the *Cauchy loss*, which has the form (3) except that $\phi$ is defined by

$$\begin{aligned} \phi (\theta ) = \frac{\xi ^2}{2} \log \left( 1+ \frac{\theta ^2}{\xi ^2} \right) , \end{aligned}$$

for some parameter $\xi$. This function is $\rho$-weakly convex with $\rho =6$.

### Complexity Bounds for Weakly Convex Problems

To put our results in perspective, we provide a review of the literature on complexity bounds for optimization problems related to our formulation (1), including weakly convex functions. In all cases, these are bounds on the number of iterations required to find an approximately stationary point, where our measure of stationarity is based the norm of the gradient of the Moreau envelope (a smooth proxy).

The best-known complexity for black box subgradient optimization for weakly convex functions is ${\mathcal {O}}(\epsilon ^{-4})$. This result is proved for *stochastic* subgradient in [22], but as in the convex case, there is no known improvement in the deterministic setting. As in convex optimization, subgradient methods are quite general and implementable for weakly convex functions. However, when more structure is present in the function, algorithms that achieve better complexity can be devised. In particular, when the proximal operator of the nonsmooth weakly convex function can be calculated analytically, complexity bounds of ${\mathcal {O}}(\epsilon ^{-2})$ can be proved (see Sect. 5), the same bounds as for steepest descent methods in the smooth nonconvex case. This means that the entire difficulty introduced by the nonsmoothness can be mitigated as long as the nonsmoothness can be treated by a proximal operator.

For convex optimization problems, bounds of ${\mathcal {O}}(\epsilon ^{-1})$ can be obtained for gradient methods on smooth functions and ${\mathcal {O}}(\epsilon ^{-1/2})$ for accelerated gradient methods. These same bounds can also be obtained for nonsmooth problems provided that the nonsmooth term is handled by a proximal operator. When the explicit proximal operator is not available and subgradient methods have to be used, the complexity reverts to ${\mathcal {O}}(\epsilon ^{-2})$.

It is possible to keep the ${\mathcal {O}}(\epsilon ^{-2})$ rate when just a local model of the weakly convex part is evaluated by a convex operator. The paper [23] studies optimization problems of the type

$$\begin{aligned} \min _x \, h(x) + g(c(x)) \end{aligned}$$

where *h* is convex, proper and closed; *g* is convex and Lipschitz continuous; and *c* is smooth. (Under these assumptions, the composition $g\circ c$ is weakly convex.) The ${\mathcal {O}}(\epsilon ^{-2})$ bound is proved for an algorithm in which the (convex) subproblem

4 $$\begin{aligned} \min _y \, h(y) + g(c(x) + \nabla c(x)(y-x)) + \frac{1}{2 t} \Vert y-x \Vert ^2 \end{aligned}$$

is solved explicitly. In the more realistic case in which (4) must be solved by an iterative procedure, a bound of $\widetilde{{\mathcal {O}}}(\epsilon ^{-3})$ is obtained in [23]. (The symbol $\widetilde{{\mathcal {O}}}$ hides logarithmic terms.)

Functions of the form *g*(*c*(*x*)) have also been studied in [24] for the case of a smooth nonlinear vector function *c* and a prox-regular *g*. This formulation is more general than those considered in this paper, both in the fact that all weakly convex functions are prox-regular, and in the nonlinearity of the inner map *c*. The subproblems in [24] have a form similar to (4), and while convergence results are proved in the latter paper, it does not contain rate-of-convergence results or complexity results.

A different weakly convex structure is explored in [25], in which the weak convexity stems from a smooth saddle point problem. This paper studies the problem

$$\begin{aligned} \min _x \, \max _{y\in Y} \, l(x,y), \end{aligned}$$

for a compact set $Y \subset {\mathbb {R}}^m$, where $l(x,\cdot )$ is concave, $l(\cdot ,y)$ is nonconvex, and $l(\cdot ,\cdot )$ is smooth. An iteration bound of $\widetilde{{\mathcal {O}}}(\epsilon ^{-3})$ is proved for a method that uses only gradient evaluations.

In light of the considerations above, the complexity bound of ${\mathcal {O}}(\epsilon ^{-3})$ for our algorithm seems almost inevitable. It interpolates between the setting without structural assumptions about the nonsmoothness (black box subgradient) and the perfect structural knowledge of the nonsmoothness (explicit knowledge of the proximal operator).

In Sect. 5, we treat the simpler setting in which the linear operator from (1) is the identity, so that $F(x) = h(x)+g(x)$. Similar problems have been analyzed before, for example, in [15, 17]. However, it is assumed in [17] that convexity in the data fidelity term *h* compensates for nonconvexity in the regularizer *g* such that the overall objective function *F* remains convex. (We make no such assumption here.) The paper [15] does not make such restrictive assumptions and proves convergence but not complexity bounds.

## Preliminaries

The concept of subgradient of a convex function can be adapted to weakly convex functions via the following definition.

### Definition 3.1

(Fréchet subdifferential) Let $g:{\mathbb {R}}^n \rightarrow {\overline{{\mathbb {R}}}}$ be a function and ${\bar{y}}$ a point such that $g({\bar{y}})$ is finite. Then, the *Fréchet subdifferential* of *g* at ${\bar{y}}$, denoted by $\partial g({\bar{y}})$, is the set of all vectors $v \in {\mathbb {R}}^n$ such that

5 $$\begin{aligned} g(y) \ge g({\bar{y}}) + \langle v, y- {\bar{y}}\rangle + o(\Vert y-{\bar{y}} \Vert ) \quad \text {as }y \rightarrow {\bar{y}}. \end{aligned}$$

Modifying the convex case, in which subgradients are the slopes of linear functions that underestimate *g* but coincide with it at ${\bar{y}}$, Fréchet subgradients do so *up to first order*. This definition makes sense for arbitrary functions, but for lower semicontinuous $\rho$-weakly convex functions, more can be said. For example, for this class of function we know that subgradients satisfy the following stronger version of (5), for all $v \in \partial g({\bar{y}})$,

$$\begin{aligned} g(y) \ge g({\bar{y}}) + \langle v, y - {\bar{y}} \rangle - \frac{\rho }{2} \Vert y-{\bar{y}} \Vert ^2, \quad \forall y \in {\mathbb {R}}^{n}. \end{aligned}$$

Further, if we assume the weakly convex function to be continuous at a point *y*, then its subdifferential is nonempty at *y*. Both of these claims can be verified directly by adding $\frac{\rho }{2}\Vert \cdot \Vert ^{2}$ to *g* and considering the convex subdifferential; see [26, Lemma 2.1].

Another nice property of weakly convex functions is that the definition of a Moreau envelope (see Definition 2.1) extends without modification to weakly convex functions, subject only to a restriction on the parameter $\mu$. The proximal operator of Definition 2.1 also extends to this setting, and this operator and the Moreau envelope fulfil the same identity as in the convex setting.

### Lemma 3.1

([27, Corollary 3.4]) Let $g: {\mathbb {R}}^n \rightarrow {\overline{{\mathbb {R}}}}$ be a proper, $\rho$-weakly convex, and lower semicontinuous function, and let $\mu \in ]0,\rho ^{-1}[$. Then, the Moreau envelope $g_\mu (\cdot )$ is continuously differentiable on ${\mathbb {R}}^n$ with gradient

$$\begin{aligned} \nabla g_\mu (y) = \frac{1}{\mu }\left( y - {\text {prox}}^{}_{\mu g}\left( y \right) \right) , \quad \text{ for } \text{ all } y \in {\mathbb {R}}^n. \end{aligned}$$

This gradient is $\max \left\{ \mu ^{-1}, \frac{\rho }{1-\rho \mu } \right\}$-Lipschitz continuous. In particular, a gradient step with respect to the Moreau envelope corresponds to a proximal step, that is,

6 $$\begin{aligned} y - \mu \nabla g_\mu (y) = {\text {prox}}^{}_{\mu g}\left( y \right) , \quad \text{ for } \text{ all } y \in {\mathbb {R}}^n. \end{aligned}$$

Lemma 3.1 not only clarifies the smoothness of the Moreau envelope, but also gives a way of computing its gradient via the prox operator. Obviously, a smooth representation whose gradient could not be computed would be of only limited usefulness from an algorithmic standpoint. The only difference between the weakly convex and convex settings is that the Moreau envelope need not be convex in the former case.

### Stationarity

We say that a point ${\bar{x}}$ is a stationary point for a function if the Fréchet subdifferential of the function contains 0 at ${\bar{x}}$. The concept of *nearly stationary* is not quite so straightforward. We motivate our approach by looking first at the simple additive composite problem, also discussed in Sect. 5, which corresponds to setting $A=I$ in (1), that is,

7 $$\begin{aligned} \min _x \, h(x) + g(x). \end{aligned}$$

Stationarity for (7) means that $0 \in \partial ( h + g)({\bar{x}})$, that is, $-\nabla h({\bar{x}}) \in \partial g({\bar{x}})$. A natural definition for $\epsilon$-approximate stationarity would thus be

8 $$\begin{aligned} \text {dist}(-\nabla h(x), \partial g(x)) \le \epsilon , \end{aligned}$$

where $\text {dist}$ denotes the distance between two sets and is given for a point $x\in {\mathbb {R}}^d$ and a set $\mathcal{A}\subset {\mathbb {R}}^d$ by $\text {dist}(x,\mathcal{A}) := \inf _{y \in \mathcal{A}} \, \{\Vert x-y \Vert \}$. However, since we are running gradient descent on the *smoothed* problem, our algorithm will naturally compute and detect points with that satisfy a threshold condition of the form

9 $$\begin{aligned} \Vert \nabla h (x) + \nabla g_\mu (x) \Vert \le \epsilon . \end{aligned}$$

The next lemma helps to clarify relationship between these two conditions.

#### Lemma 3.2

Let $g: {\mathbb {R}}^n \rightarrow {\overline{{\mathbb {R}}}}$ be a proper, $\rho$-weakly convex, and lower semicontinuous function; and let $\mu \in ]0,\rho ^{-1}[$. Then,

10 $$\begin{aligned} \nabla g_\mu (x) \in \partial g ({\text {prox}}^{}_{\mu g}\left( x \right) ). \end{aligned}$$

#### Proof

From Definition 2.1, we have that

$$\begin{aligned} 0 \in \partial g({\text {prox}}^{}_{\mu g}\left( x \right) ) + \frac{1}{\mu } ({\text {prox}}^{}_{\mu g}\left( x \right) - x), \end{aligned}$$

from which the result follows when we use (6).$\square$

(This result is proved for the case of *g* convex in [23, Lemma 2.1], with essentially the same proof.)

This lemma tells us that when (9) holds, then (8) is nearly satisfied, except that in the argument of $\partial g$, *x* is replaced by ${\text {prox}}^{}_{\mu g}\left( x \right)$. In general, however, ${\text {prox}}^{}_{\mu g}\left( x \right)$ might be arbitrarily far away from *x*. We can remedy this issue by requiring *g* to be Lipschitz continuous also.

#### Lemma 3.3

Let $g:{\mathbb {R}}^n \rightarrow {\mathbb {R}}$ be a $\rho$-weakly convex function that is $L_g$-Lipschitz continuous, and let $\mu \in ]0,\rho ^{-1}[$. Then, the Moreau envelope $g_{\mu }$ is Lipschitz continuous with

11 $$\begin{aligned} \Vert \nabla g_\mu (x) \Vert \le L_g \end{aligned}$$

and

12 $$\begin{aligned} \Vert x - {\text {prox}}^{}_{\mu g}\left( x \right) \Vert \le \mu L_g, \quad \forall x\in {\mathbb {R}}^{n}. \end{aligned}$$

#### Proof

Lipschitz continuity is equivalent to bounded subgradients [28], so by (10), we have for all $x\in {\mathbb {R}}^{n}$

$$\begin{aligned} \Vert \nabla g_\mu (x) \Vert \le \sup \left\{ \Vert v \Vert \, : \, v \in \partial g({\text {prox}}^{}_{\mu g}\left( x \right) )\right\} \le L_g, \end{aligned}$$

proving (11). The bound (12) follows immediately when we use the fact that $x - {\text {prox}}^{}_{\mu g}\left( x \right) = \mu \nabla g_\mu (x)$ from Lemma 3.1.$\square$

When $x\in {\mathbb {R}}^{n}$ satisfies (9), $\nabla h$ is $L_{\nabla h}$-Lipschitz continuous, *g* is $L_{g}$ Lipschitz continuous, we have

$$\begin{aligned} \begin{aligned} \text {dist}&(-\nabla h({\text {prox}}^{}_{\mu g}\left( x \right) ), \partial g({\text {prox}}^{}_{\mu g}\left( x \right) )) \;\; \\&\le \Vert \nabla h({\text {prox}}^{}_{\mu g}\left( x \right) ) - \nabla h(x) \Vert + \text {dist}(-\nabla h(x), \partial g({\text {prox}}^{}_{\mu g}\left( x \right) )) \\&\le L_{\nabla h}\Vert x- {\text {prox}}^{}_{\mu g}\left( x \right) \Vert + \epsilon \qquad \text{(from }~(9) \hbox { and}~(10))\\&\le L_{\nabla h}L_g\mu + \epsilon \qquad \text{(from }~(12)). \end{aligned} \end{aligned}$$

Thus, if $\mu$ is sufficiently small and *x* satisfies (9), then ${\text {prox}}^{}_{\mu g}\left( x \right)$ is near-stationary for (7).

### Stationarity for the Composite Problem

It follows immediately from (10) in Lemma 3.2 that for $\mu \in ]0,\rho ^{-1}[$, we have for all $x\in {\mathbb {R}}^d$

13 $$\begin{aligned} \nabla (g_\mu \circ A)(x) = A^*\nabla g_\mu (Ax) \in A^*\partial g({\text {prox}}^{}_{\mu g}\left( Ax \right) ). \end{aligned}$$

Extending the results of the previous section to the case of a general linear operator *A* in (1) requires some work. Stationarity for (1) requires that $0 \in \nabla h(x) + A^*\partial g(Ax)$, so $\epsilon$-near stationarity requires

14 $$\begin{aligned} \text {dist}(-\nabla h(x), A^*\partial g(Ax)) \le \epsilon . \end{aligned}$$

Our method can compute a point *x* such that

$$\begin{aligned} \left\| \nabla h(x) + \nabla (g_\mu \circ A)(x) \right\| \le \epsilon \end{aligned}$$

which by (13) implies that

15 $$\begin{aligned} \text {dist}( -\nabla h(x), A^*\partial g(z)) \le \epsilon , \quad \text{ where } \;\; z={\text {prox}}^{}_{\mu g}\left( Ax \right) , \end{aligned}$$

where provided that *g* is $L_g$-Lipschitz continuous, we have

16 $$\begin{aligned} \Vert Ax - z \Vert \le L_g\mu . \end{aligned}$$

The bound in (15) measures the criticality, while the bound in (16) concerns feasibility. The bounds (15), (16) are not a perfect match with (14), since the subdifferentials of *h* and $g \circ A$ are evaluated at different points.

*Surjectivity of* ${\mathbf {A}}$. When *A* is surjective, we can perturb the *x* that satisfies (15), (16) to a nearby point $x^*$ that satisfies a bound of the form (14). Since $z={\text {prox}}^{}_{\mu g}\left( Ax \right)$ is in the range of *A*, we can define

17 $$\begin{aligned} x^* := {\mathop {{{\,\mathrm{arg\,min}\,}}}\limits _{x'\in {\mathbb {R}}^{d}}} \{\Vert x - x' \Vert ^2 \; : \; Ax' = z\}, \end{aligned}$$

which is given explicitly by

$$\begin{aligned} x^{*} = x - A^{*} {\left( AA^{*} \right) }^{-1}(Ax-z) = x- A^\dagger (Ax-z) \end{aligned}$$

where $A^\dagger := A^{*}{(AA^{*})}^{-1}$ is the pseudoinverse of *A*. The operator norm of the pseudoinverse is bounded by the inverse of the smallest singular value $\sigma _{\min }(A)$ of *A*, so when *g* is $L_g$-Lipschitz continuous, we have from (16) that

18 $$\begin{aligned} \Vert x - x^* \Vert \le {\sigma _{\min }(A)}^{-1} \Vert Ax-z\Vert \le {\sigma _{\min }(A)}^{-1} L_{g}\mu . \end{aligned}$$

The point $x^{*}$ is approximately stationary in the sense of (14), for $\mu$ sufficiently small, because

19 $$\begin{aligned} \text {dist}&(-\nabla h(x^{*}), A^* \partial g(Ax^{*}))&\;\; \nonumber \\&\le \Vert \nabla h(x^{*}) - \nabla h(x) \Vert + \text {dist}(-\nabla h(x), A^* \partial g(z))&\;\; \text{(since } Ax^{*}=z\hbox {)} \nonumber \\&\le L_{\nabla h}\Vert x- x^{*} \Vert + \epsilon&\;\; \text{(from }~(15)) \nonumber \\&\le L_{\nabla h}{\sigma _{\min }(A)}^{-1}L_g\mu + \epsilon&\;\; \text{(from }~(18)). \end{aligned}$$

By choosing $\mu$ small, $x^{*}$ will be an approximate solution in the stronger sense (14) and not just the weaker notion of (15), (16), which is the case if *A* is not surjective.

## Variable Smoothing

We describe our variable smoothing approaches for the problem (1), where we assume that *h* is $L_{\nabla h}$-smooth; *g* is possibly nonsmooth, $\rho$-weakly convex, and $L_g$-Lipschitz continuous; and *A* is a nonzero linear continuous operator. For convenience, we define the smoothed approximation $F_{k}:{\mathbb {R}}^{d} \rightarrow {\mathbb {R}}$ based on the Moreau envelope with parameter $\mu _k$ as follows:

$$\begin{aligned} F_k(x) := h(x) + g_{\mu _k} (Ax). \end{aligned}$$

We note from Lemma 3.1 and the chain rule that

20 $$\begin{aligned} \nabla F_k(x) = \nabla h(x) + \frac{1}{\mu _k} A^* (Ax-{\text {prox}}^{}_{\mu _k g}\left( Ax \right) ). \end{aligned}$$

The quantity $L_k$ defined by

21 $$\begin{aligned} L_k := L_{\nabla h} + \Vert A \Vert ^2 \max \left\{ \mu ^{-1}_{k}, \frac{\rho }{1-\rho \mu _{k}} \right\} \end{aligned}$$

is a Lipschitz constant of the gradient of $\nabla F_k$; see Lemma 3.1. When $\rho \mu _k \le 1/2$, the maximum in (21) is achieved by $\mu _k^{-1}$, so in this case we can define

22 $$\begin{aligned} L_k := L_{\nabla h} + \Vert A \Vert ^2/\mu _{k}. \end{aligned}$$

### An Elementary Approach

Our first algorithm takes gradient descent steps on the smoothed problem, that is,

23 $$\begin{aligned} x_{k+1} = x_k - \gamma _k \nabla F_k(x_k), \end{aligned}$$

for certain values of the parameter $\mu _k$ and step size $\gamma _k$. From (20), the formula (23) is equivalent to

$$\begin{aligned} x_{k+1} = x_k - \frac{\gamma _k}{\mu _k}A^*(Ax_k - {\text {prox}}^{}_{\mu _k g}\left( Ax_k \right) ) - \gamma _k \nabla h(x_k). \end{aligned}$$

Our basic algorithm is described next.
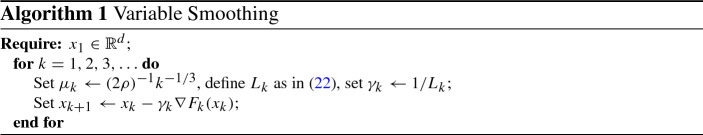


We now state the convergence result for Algorithm 1. This result and later results make use of a quantity

24 $$\begin{aligned} F^* := \liminf _{k \rightarrow \infty } F_k(x_k), \end{aligned}$$

which is finite if *F* is bounded below (and possibly in other circumstances too). (When $F^* = -\infty$, the claim of the theorem is vacuously true.) We also make use of the following quantity:

25 $$\begin{aligned} x^{*}_j:= x_j - A^{\dagger }(Ax_j-{\text {prox}}^{}_{\mu _jg}\left( Ax_j \right) ). \end{aligned}$$

#### Theorem 4.1

Suppose that Algorithm 1 is applied to the problem (1), where *g* is $\rho$-weakly convex and $\nabla h$ and *g* are Lipschitz continuous with constants $L_{\nabla h}$ and $L_g$, respectively. We have

$$\begin{aligned} \begin{aligned} \min _{1\le j \le k} \, \text {dist}( -\nabla h(x_j),&A^*\partial g({\text {prox}}^{}_{\mu _j g}\left( Ax_j \right) )) \\ \le&k^{-1/3} \sqrt{L_{\nabla h} + 2\rho \Vert A \Vert ^2}\sqrt{F_1(x_1) - F^* + {(2\rho )}^{-1} L_g^2}, \end{aligned} \end{aligned}$$

where

$$\begin{aligned} \Vert Ax_j - {\text {prox}}^{}_{\mu _j g}\left( Ax_j \right) \Vert \le j^{-1/3} {(2\rho )}^{-1} L_g, \end{aligned}$$

and $F^*$ is defined as in (24). If *A* is also surjective, then for $x_k^*$ defined as in (25), we have

$$\begin{aligned} \begin{aligned} \min _{1\le j \le k} \,&\text {dist}( -\nabla h(x^{*}_j), A^*\partial g(Ax_j^{*})) \\ \le&k^{-1/3} \bigg (\sqrt{L_{\nabla h} + 2\rho \Vert A \Vert ^2}\sqrt{F_1(x_1) - F^* + {(2\rho )}^{-1} L_g^2} + L_{\nabla h} {\sigma _{\min }(A)}^{-1}L_{g}\bigg ) \end{aligned} \end{aligned}$$

and $$\Vert x_{j}-x_{j}^{*} \Vert \le {\sigma _{\min }(A)}^{-1}L_{g}\mu _{j} = {\sigma _{\min }(A)}^{-1}L_{g} {(2\rho )}^{-1} j^{-1/3}$$.

Before proving this theorem, we state and prove a lemma that relates the function values of two Moreau envelopes with two different smoothing parameters. In the convex case, such statements are well known, but in the nonconvex case this result is novel.

#### Lemma 4.1

Let $g: {\mathbb {R}}^n \rightarrow {\overline{{\mathbb {R}}}}$ be a proper, closed, and $\rho$-weakly convex function, and let $\mu _2$ and $\mu _1$ be parameters such that $0< \mu _2 \le \mu _1 < \rho ^{-1}$. Then, we have

$$\begin{aligned} g_{\mu _2}(y) \le g_{\mu _1}(y) + \frac{1}{2} \frac{\mu _1 - \mu _2}{\mu _2}\mu _1\Vert \nabla g_{\mu _1}(y) \Vert ^2. \end{aligned}$$

If, in addition, *g* is $L_g$-Lipschitz continuous, we have

$$\begin{aligned} g_{\mu _2}(y) \le g_{\mu _1}(y) + \frac{1}{2} \frac{\mu _1 - \mu _2}{\mu _2}\mu _1 L_g^2. \end{aligned}$$

#### Proof

By using the definition of the Moreau envelope, together with Lemma 3.1, we obtain

$$\begin{aligned} \begin{aligned}&g_{\mu _2}(y) \\&\quad = \min _{u\in {\mathbb {R}}^n} \, \left\{ g(u) + \frac{1}{2 \mu _2}\Vert y-u \Vert ^2\right\} \\&\quad = \min _{u\in {\mathbb {R}}^n} \, \left\{ g(u) + \frac{1}{2 \mu _1}\Vert y-u \Vert ^2 + \frac{1}{2}\left( \frac{1}{\mu _2} - \frac{1}{\mu _1}\right) \Vert y-u \Vert ^2\right\} \\&\quad \le g({\text {prox}}^{}_{\mu _1 g}\left( y \right) ) + \frac{1}{2 \mu _1}\Vert y-{\text {prox}}^{}_{\mu _1 g}\left( y \right) \Vert ^2 + \frac{1}{2}\left( \frac{1}{\mu _2} - \frac{1}{\mu _1}\right) \Vert y-{\text {prox}}^{}_{\mu _1 g}\left( y \right) \Vert ^2 \\&\quad = g_{\mu _1}(y) + \frac{1}{2}\left( \frac{\mu _1-\mu _2}{\mu _2}\right) \mu _1 \Vert \nabla g_{\mu _1} (y) \Vert ^2, \end{aligned} \end{aligned}$$

proving the first claim. The second claim follows immediately from (11).$\square$

#### Proof of Theorem 4.1

Since $L_k = 1/\gamma _k$ is the Lipschitz constant of $\nabla F_k$, we have for any $k=1,2, \ldots$ that

$$\begin{aligned} F_k(x_{k+1}) \le F_k(x_k) + \langle \nabla F_k(x_k), x_{k+1} - x_k\rangle + \frac{1}{2 \gamma _k} \Vert x_{k+1} - x_k \Vert ^2. \end{aligned}$$

By substituting the definition of $x_{k+1}$ from (23), we have

26 $$\begin{aligned} F_k(x_{k+1}) \le F_k(x_k) - \frac{\gamma _k}{2} \Vert \nabla F_k(x_k) \Vert ^2. \end{aligned}$$

From Lemma 4.1, we have for all $x \in {\mathbb {R}}^{d}$

$$\begin{aligned} F_{k+1}(x)\le F_k(x) + \frac{1}{2}(\mu _k - \mu _{k+1})\frac{\mu _k}{\mu _{k+1}}\Vert (\nabla g_{\mu _k})(Ax)\Vert ^2 \le F_k(x) + (\mu _k - \mu _{k+1}) L_g^2, \end{aligned}$$

where we used in the second inequality that $\frac{\mu _k}{\mu _{k+1}} \le 2$. We set $x=x_{k+1}$ and substitute into (26) to obtain

$$\begin{aligned} F_{k+1}(x_{k+1}) \le F_k(x_k) - \frac{\gamma _k}{2} \Vert \nabla F_k(x_k) \Vert ^2 + (\mu _k - \mu _{k+1})L_g^2. \end{aligned}$$

By summing both sides of this expression over $k=1,2, \ldots K$, and telescoping, we deduce that

27 $$\begin{aligned} \sum _{k=1}^K \frac{\gamma _k}{2} \Vert \nabla F_k(x_k) \Vert ^2&\le F_1(x_1) - F_K(x_K) + (\mu _1 - \mu _K)L_g^2 \nonumber \\&\le F_1(x_1) - F^* + \mu _1L_g^2. \end{aligned}$$

Since

$$\begin{aligned} \gamma _k = \frac{\mu _k}{\mu _k L_{\nabla h} + \Vert A \Vert ^2}\ge k^{-1/3}\frac{{(2\rho )}^{-1}}{{(2\rho )}^{-1}L_{\nabla h}+ \Vert A \Vert ^2} = k^{-1/3}\frac{1}{L_{\nabla h}+ 2 \rho \Vert A \Vert ^2}. \end{aligned}$$

we have from (27) that

28 $$\begin{aligned} \frac{1}{L_{\nabla h}+ 2 \rho \Vert A \Vert ^2} \min _{1 \le j \le K} \, \Vert \nabla F_j(x_j) \Vert ^2 \frac{1}{2} \sum _{k=1}^K k^{-1/3} \le F_1(x_1) - F^* + {(2\rho )}^{-1}L_g^2. \end{aligned}$$

Now we observe that

$$\begin{aligned} \begin{aligned} \sum _{k=1}^{K} k^{-1/3}&\ge \sum _{k=1}^{K} \int _{k}^{k+1} x^{-1/3} \mathop {}\!\mathrm {d}x = \int _{1}^{K+1} x^{-1/3} \mathop {}\!\mathrm {d}x = \frac{3}{2} \left( {(K+1)}^{2/3} -1\right) \\&\ge {(K+1)}^{2/3} - 1 \ge \frac{1}{2} K^{2/3}, \quad K=1,2, \ldots \end{aligned} \end{aligned}$$

where the final inequality can be checked numerically. Therefore, by substituting into (28), we have

$$\begin{aligned} \min _{1 \le j \le K} \, \Vert \nabla F_j(x_j) \Vert ^2 \le 4\frac{L_{\nabla h}+ (2\rho )\Vert A \Vert ^2}{K^{2/3}} \Big (F_1(x_1) - F^* + {(2\rho )}^{-1}L_g^2\Big ), \end{aligned}$$

and so

29 $$\begin{aligned} \min _{1 \le j \le K} \, \Vert \nabla F_j(x_j) \Vert \le \frac{C}{K^{1/3}}, \end{aligned}$$

where $C:= 2\sqrt{L_{\nabla h}+ (2\rho )\Vert A \Vert ^2} \sqrt{F_1(x_1) - F^* + {(2\rho )}^{-1} L_g^2}$. By combining this bound with (15), and defining $z_j := {\text {prox}}^{}_{\mu _j g}\left( Ax_j \right)$, we obtain

30 $$\begin{aligned} \min _{1\le j \le k} \, \text {dist}(-\nabla h(x_j), A^*\partial g(z_j) ) \le \min _{1 \le j \le k} \, \Vert \nabla F_j(x_j) \Vert \le \frac{C}{k^{1/3}}, \end{aligned}$$

where we deduce from (12) that

31 $$\begin{aligned} \Vert Ax_j - z_j \Vert \le \frac{{(2\rho )}^{-1}L_g}{j^{1/3}} , \quad \text{ for } \text{ all }\, j \ge 1. \end{aligned}$$

The second statement concerning surjectivity of *A* follows from the consideration made in (17) to (19).$\square$

There is a mismatch between the two bounds in this theorem. The first bound (the criticality bound) indicates that during the first $k = O(\epsilon ^{-3})$ iterations, we will encounter an iteration *j* at which the first-order optimality condition is satisfied to within a tolerance of $\epsilon$. However, this bound could have been satisfied at an early iteration (that is, $j \ll \epsilon ^{-3}$), for which value the second (feasibility) bound, on $\Vert Ax_j - {\text {prox}}^{}_{\mu _j g}\left( Ax_j \right) \Vert$, may not be particularly small. The next section describes an algorithm that remedies this defect.

### An Epoch-Wise Approach with Improved Convergence Guarantees

We describe a variant of Algorithm 1 in which the steps are organized into a series of epochs, each of which is twice as long as the one before. We show that there is some iteration $j = O(\epsilon ^{-3})$ such that both $\Vert Ax_j - {\text {prox}}^{}_{\mu _j g}\left( Ax_j \right) \Vert$ and $\text {dist}( -\nabla h(x_j), A^*\partial g({\text {prox}}^{}_{\mu _j g}\left( Ax_j \right) ))$ are smaller than the given tolerance $\epsilon$.
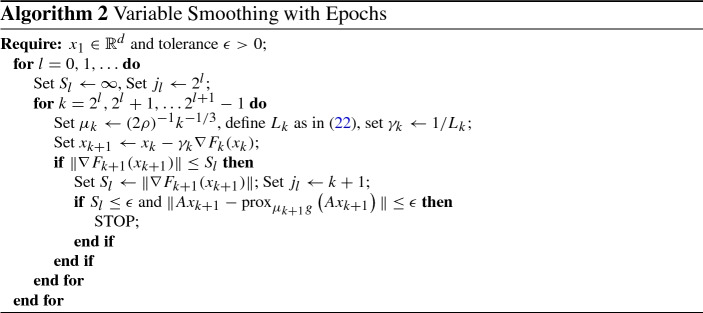


#### Theorem 4.2

Consider solving (1) using Algorithm 2, where *h* and *g* satisfy the assumptions of Theorem 4.1 and $F^*$ defined in (24) is finite. For a given tolerance $\epsilon >0$, Algorithm 2 generates an iterate $x_j$ for some $j = O(\epsilon ^{-3})$ such that

$$\begin{aligned} \text {dist}( -\nabla h(x_j), A^*\partial g(z_j)) \le \epsilon \quad \text {and} \quad \Vert Ax_j - z_j \Vert \le \epsilon , \end{aligned}$$

where $$z_j = {\text {prox}}^{}_{\mu _{j} g}\left( Ax_{j} \right) $$.

#### Proof

As in (27), by using monotonicity of $\{ F_k(x_k) \}$ and discarding nonnegative terms, we have that

$$\begin{aligned} \sum _{k=2^l}^{2^{l+1}-1} \frac{\gamma _k}{2} \Vert \nabla F_k(x_k) \Vert ^2 \le F_1(x_1) - F^* + {(2\rho )}^{-1} L_g^2. \end{aligned}$$

With the same arguments as in the earlier proof, we obtain

$$\begin{aligned} \begin{aligned} \sum _{k=2^l}^{2^{l+1}-1} k^{-1/3}&\ge \sum _{k=2^l}^{2^{l+1}-1} \int _k^{k+1} x^{-1/3} \mathop {}\!\mathrm {d}x = \int _{2^l}^{2^{l+1}} x^{-1/3} \mathop {}\!\mathrm {d}x \\&= \frac{3}{2} \left( {(2^{l+1})}^{2/3} - {(2^{l})}^{2/3} \right) = \frac{3}{2} \left( 2^{2/3}-1\right) {(2^l)}^{2/3} \ge \frac{1}{2} {(2^l)}^{2/3}. \end{aligned} \end{aligned}$$

Therefore, we have

$$\begin{aligned} \min _{2^l \le j \le 2^{l+1}-1} \, \Vert \nabla F_j(x_j) \Vert \le \frac{C}{{(2^{l})}^{1/3}}, \end{aligned}$$

with $C=2\sqrt{L_{\nabla h}+ 2\rho \Vert A \Vert ^2}\sqrt{ F_1(x_1) - F^* + {(2\rho )}^{-1} L_g^2}$ as before. Noting that $z_j := {\text {prox}}^{}_{\mu _j g}\left( Ax_j \right)$, we have as in (30) that

32 $$\begin{aligned} \min _{2^l \le j \le 2^{l+1}-1} \, \text {dist}( -\nabla h(x_j), A^*\partial g(z_j) ) \le \frac{C}{{(2^l)}^{1/3}}, \end{aligned}$$

as previously. Further, we have for $2^l \le j \le 2^{l+1}-1$ that

33 $$\begin{aligned} \Vert Ax_j - z_j \Vert \le L_g \mu \le \frac{{(2\rho )}^{-1}L_g}{j^{1/3}} \le \frac{{(2\rho )}^{-1}L_g}{{(2^l)}^{1/3}}. \end{aligned}$$

From (32) and (33), we deduce that Algorithm 2 must terminate before the end of epoch *l*, that is, before $2^{l+1}$ iterations have been completed, where *l* is the first nonnegative integer such that

$$\begin{aligned} 2^l \ge \max \{C^3, {(2\rho )}^{-3} L_g^3\}\epsilon ^{-3}. \end{aligned}$$

Thus, termination occurs after at most $2 \max \{C^3, {(2\rho )}^{-3} L_g^3\}\epsilon ^{-3}$ iterations.$\square$

For the case of *A* surjective, we have the following stronger result.

#### Corollary 4.1

Suppose that the assumptions of Theorem 4.2 hold, that *A* is also surjective, and that the condition $\Vert Ax_{k+1} - {\text {prox}}^{}_{\mu _{k+1} g}\left( Ax_{k+1} \right) \Vert \le \epsilon$ in Algorithm 2 is replaced by $\Vert x_{k+1}-x_{k+1}^{*} \Vert \le \epsilon$, where $x_{k+1}^*$ is defined in (25). Then, for some $j'=O(\epsilon ^{-3})$, we have that

$$\begin{aligned} \text {dist}\, ( -\nabla h(x^{*}_{j'}), A^*\partial g(Ax_{j'}^{*})) \le \epsilon \end{aligned}$$

and $$\Vert x_{j'}-x_{j'}^{*} \Vert \le \epsilon $$.

#### Proof

With the considerations made in the previous proof as well as the one made in (17) to (19), we can choose *l* to be the smallest positive integer such that

$$\begin{aligned} 2^{l+1} \ge 2\max \{C^{3}, {\sigma _{\min }(A)}^{-3}L_{g}^{3}{(2\rho )}^{-3}\}\epsilon ^{-3}. \end{aligned}$$

The claim then holds for some $j' \le 2^{l+1}$.$\square$

Although Algorithm 2 seems more complicated than Algorithm 1, the steps are the same. The only difference is that for the second algorithm, we do not search for the iterate that minimizes criticality across *all* iterations but only across at most the last *k*/2 iterations, where *k* is the total number of iterations.

#### Remark 4.1

For both versions of our proposed method we use an explicit choice of smoothing parameters, choosing $\mu _k$ to be a multiple of ${\mathcal {O}}(k^{-1/3})$. This specific dependence on *k* achieves a balance between criticality and feasibility. As can be seen from (29) (criticality measure) and (31) (feasibility measure) both measures decrease like $k^{-1/3}$. A slower decrease in $\mu _k$ would result in a faster decrease in the criticality measure but a slower decrease in the feasibility measure—and vice versa.

#### Remark 4.2

Our technique does not adapt in an obvious way to the case in which *g* is actually convex. Typically, we know in advance whether or not *h* and *g* in (1) are convex, and if they are, we could choose one of the well-established methods that make use of gradients, proximal operators, and possibly acceleration. See, for example the proximal accelerated gradient approach of [3], which achieves a rate of ${\mathcal {O}}(k^{-1})$. A method in the spirit of [29], which automatically adapts to convexity and simultaneously achieves the optimal rates for both nonconvex and convex problems would be desirable, but is outside the scope of this work.

## Proximal Gradient

Here we derive a complexity bound for the proximal gradient algorithm applied to the more elementary problem (7) studied in Sect. 3.1, that is,

34 $$\begin{aligned} \min _{x\in {\mathbb {R}}^{d}} \, F(x) := h(x) + g(x), \end{aligned}$$

for $h:{\mathbb {R}}^{d}\rightarrow {\mathbb {R}}$ a $L_{\nabla h}$-smooth function and $g:{\mathbb {R}}^d \rightarrow {\overline{{\mathbb {R}}}}$ a possibly nonsmooth, $\rho$-weakly convex function. Such a bound has not been made explicit before, to the authors’ knowledge, though it is a fairly straightforward consequence of existing results. The bound makes an interesting comparison with the result in Sect. 4, where the nonsmoothness issue becomes more complicated due to the composition with a linear operator. In this section, we assume that a closed-form proximal operator is available for *g*, and we show that the complexity bound of ${\mathcal {O}}(\epsilon ^{-2})$ is the same order as for gradient descent applied to smooth nonconvex functions.

Standard proximal gradient applied to problem (34), for a given step size $\lambda \in ]0,\min \{\rho ^{-1}/2, L_{\nabla h}^{-1}\}]$ and initial point $x_1$, is as follows:

35 $$\begin{aligned} x_{k+1}&:= \arg \min _{x\in {\mathbb {R}}^d} \left\{ g(x) + \langle \nabla h(x_k), x - x_k\rangle +\frac{1}{2 \lambda } \Vert x-x_k \Vert ^2 \right\} , \nonumber \\&= {\text {prox}}^{}_{\lambda g}\left( x_k - \lambda \nabla h(x_k) \right) , \quad k=1,2, \ldots \end{aligned}$$

where the choice of $\lambda$ ensures that the function to be minimized in (35) is $(\lambda ^{-1}-\rho )$-strongly convex, so that $x_{k+1}$ is uniquely defined.

We have the following convergence result.

### Theorem 5.1

Consider the algorithm defined by (35) applied to problem (34), where we assume that *g* is proper, lower semicontinuous and $\rho$-weakly convex and that $\nabla h$ is Lipschitz continuous with constant $L_{\nabla h}$. Supposing that the step size $\lambda \in ]0,\min \{\rho ^{-1}/2, L_{\nabla h}^{-1}\}]$, we have for all $k \ge 1$ that

$$\begin{aligned} \min _{2\le j\le k+1} \, \text {dist}(0, \partial (h+g)(x_j)) \le k^{-1/2} \sqrt{2(F(x_1)-F^*)} \; \frac{\lambda ^{-1}+L_{\nabla h}}{\sqrt{\lambda ^{-1}-\rho }}, \end{aligned}$$

where $F^*$ is defined in (24).

### Proof

Note first that the result is vacuous if $F^*=-\infty$, so we assume henceforth that $F^*$ is finite. We have for every $x \in {\mathbb {R}}^d$ that

$$\begin{aligned}&g(x_{k+1}) {+} h(x_{k}) {+} \langle \nabla h(x_{k}), x_{k+1}{-}x_{k}\rangle + \frac{1}{2 \lambda }\Vert x_{k+1}{-}x_{k} \Vert ^2 {+} \frac{1}{2} (\lambda ^{-1} - \rho ) \Vert x - x_{k+1} \Vert ^2\\&\quad \le g(x) + h(x_k) + \langle \nabla h(x_k), x-x_k\rangle + \frac{1}{2 \lambda } \Vert x - x_k \Vert ^2. \end{aligned}$$

By applying the inequality

$$\begin{aligned} h(x_{k+1}) \le h(x_{k}) + \langle \nabla h(x_{k}), x_{k+1}-x_{k}\rangle + \frac{1}{2 \lambda }\Vert x_{k+1}-x_{k} \Vert ^2, \quad \text{ for } \text{ all } x \in {\mathbb {R}}^d, \end{aligned}$$

obtained from the Lipschitz continuity of $\nabla h$ and the fact that $\lambda \le L_{\nabla h}^{-1}$, we deduce that

$$\begin{aligned} F(x_{k+1}) + \frac{1}{2} (\lambda ^{-1} - \rho ) \Vert x - x_{k+1} \Vert ^2 \le g(x) + h(x_k) + \langle \nabla h(x_k), x-x_k\rangle + \frac{1}{2 \lambda } \Vert x - x_k \Vert ^2, \end{aligned}$$

for every $x \in {\mathbb {R}}^d$. By setting $x=x_k$, we obtain

$$\begin{aligned} F(x_{k+1}) + \frac{1}{2} (\lambda ^{-1} - \rho ) \Vert x_k - x_{k+1} \Vert ^2 \le F(x_k), \end{aligned}$$

which shows, together with the definition (24), that

36 $$\begin{aligned} \sum _{k=1}^\infty \Vert x_k - x_{k+1} \Vert ^2 \le \frac{2(F(x_1)-F^*)}{\lambda ^{-1}-\rho }. \end{aligned}$$

From the optimality conditions for (35), we obtain

$$\begin{aligned} 0 \in \nabla h(x_k) + \partial g(x_{k+1}) + \lambda ^{-1}(x_{k+1}-x_k) \end{aligned}$$

which also shows that

37 $$\begin{aligned} w_{k+1} := \frac{1}{\lambda } (x_k - x_{k+1}) + \nabla h(x_{k+1}) - \nabla h(x_k) \in \partial (h+g)(x_{k+1}), \end{aligned}$$

so that

$$\begin{aligned} \Vert w_{k+1} \Vert ^2 \le {(\lambda ^{-1}+L_{\nabla h})}^2 \Vert x_k-x_{k+1}\Vert ^2. \end{aligned}$$

By combining this bound with (36), we obtain

$$\begin{aligned} \sum _{k=1}^{\infty } \Vert w_{k+1}\Vert ^2 \le 2(F(x_1)-F^*) \frac{{(\lambda ^{-1}+L_{\nabla h})}^2}{\lambda ^{-1}-\rho }. \end{aligned}$$

from which it follows that

$$\begin{aligned} \min _{1\le j\le k} \Vert w_{j+1} \Vert \le \sqrt{2 (F(x_1)-F^*)} \; \frac{(\lambda ^{-1}+L_{\nabla h})}{\sqrt{\lambda ^{-1}-\rho }}. \end{aligned}$$

The result now follows from (37), when we note that

$$\begin{aligned} \min _{1\le j\le k} \, \text {dist}(0, \partial (h+g)(x_{j+1})) \le \min _{1\le j\le k} \, \Vert w_{j+1} \Vert . \square \end{aligned}$$

This theorem indicates that the proximal gradient algorithm requires at most ${\mathcal {O}}(\epsilon ^{-2})$ to find an iterate with $\epsilon$-approximate stationarity. This bound contrasts with the bound ${\mathcal {O}}(\epsilon ^{-3})$ of Sect. 4 for the case of general *A*. Moreover, the ${\mathcal {O}}(\epsilon ^{-2})$ bound has the same order as the bound for gradient descent applied to general smooth nonconvex optimization.

## Conclusions

We consider a standard problem formulation in which a linear transformation of the input variables is composed with a nonsmooth regularizer and added to a smooth function. In most works, the regularizer is assumed to be convex, but we extend here to the case in which it is only weakly convex. This extension allows for functions which introduce desired properties, such as sparsity, without causing a bias. [Two examples from robust statistics are minimax concave penalty (MCP) and smoothly clipped absolute deviation (SCAD)], We propose a novel method based on the variable smoothing framework and show a complexity of ${\mathcal {O}}(\epsilon ^{-3})$ to obtain an $\epsilon$-approximate solution. This iteration complexity falls strictly between the iteration complexity of smooth (nonconvex) problems (${\mathcal {O}}(\epsilon ^{-2})$) and that of the black box subgradient method for weakly convex function (${\mathcal {O}}(\epsilon ^{-4})$) which assumes no knowledge of the structure of the nonsmoothness.

**Fig. 1:**
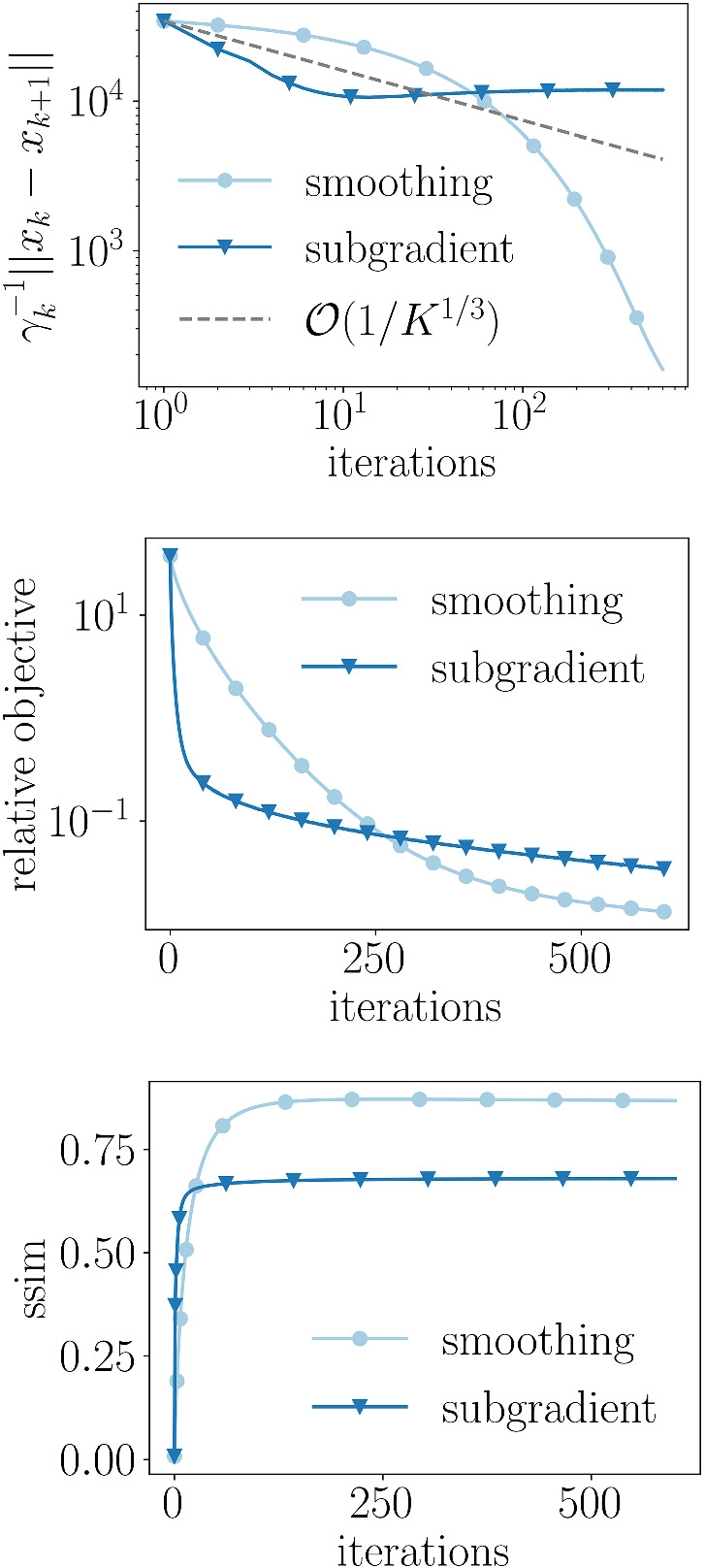
Progress of our smoothing algorithm and a naive subgradient algorithm on an image denoising problem, where minimax concave penalty is used instead of the 1-norm in the anisotropic total variation, showing better performance by the smoothing approach. **Top:** The difference of consecutive iterates scaled by the inverse of the stepsize, representing the norm of the (sub)gradient used at each iteration. **Middle:** Relative difference between the objective function at the current iterate and an approximate minimum. **Bottom:** The quality of the resulting reconstruction measured via the *structural similarity index measure*, see [30]

A performance comparison between our smoothed approach and the black-box subgradient algorithm on an image denoising problem that uses an MCP total variation regularizer is shown in Figs.1.
